# Overcoming CRISPR-Cas9 off-target prediction hurdles: A novel approach with ESB rebalancing strategy and CRISPR-MCA model

**DOI:** 10.1371/journal.pcbi.1012340

**Published:** 2024-09-03

**Authors:** Yanpeng Yang, Yanyi Zheng, Quan Zou, Jian Li, Hailin Feng

**Affiliations:** 1 School of Mathematics and Computer science, Zhejiang A&F University, Hangzhou, China; 2 College of Landscape Architecture, Beijing Forestry University, Beijing, China; 3 Institute of Fundamental and Frontier Sciences, University of Electronic Science and Technology of China, Chengdu, China; 4 Yangtze Delta Region Institute (Quzhou), University of Electronic Science and Technology of China, Quzhou, China; Indian Institute of Technology Mandi - Kamand Campus: Indian Institute of Technology Mandi, INDIA

## Abstract

The off-target activities within the CRISPR-Cas9 system remains a formidable barrier to its broader application and development. Recent advancements have highlighted the potential of deep learning models in predicting these off-target effects, yet they encounter significant hurdles including imbalances within datasets and the intricacies associated with encoding schemes and model architectures. To surmount these challenges, our study innovatively introduces an Efficiency and Specificity-Based (ESB) class rebalancing strategy, specifically devised for datasets featuring mismatches-only off-target instances, marking a pioneering approach in this realm. Furthermore, through a meticulous evaluation of various One-hot encoding schemes alongside numerous hybrid neural network models, we discern that encoding and models of moderate complexity ideally balance performance and efficiency. On this foundation, we advance a novel hybrid model, the CRISPR-MCA, which capitalizes on multi-feature extraction to enhance predictive accuracy. The empirical results affirm that the ESB class rebalancing strategy surpasses five conventional methods in addressing extreme dataset imbalances, demonstrating superior efficacy and broader applicability across diverse models. Notably, the CRISPR-MCA model excels in off-target effect prediction across four distinct mismatches-only datasets and significantly outperforms contemporary state-of-the-art models in datasets comprising both mismatches and indels. In summation, the CRISPR-MCA model, coupled with the ESB rebalancing strategy, offers profound insights and a robust framework for future explorations in this field.

## Author summary

In the field of gene editing, the application of deep learning technologies holds significant promise for predicting off-target effects in the CRISPR-Cas9 system. Nevertheless, one of the primary challenges encountered is the extreme imbalance among classes within the off-target datasets, which severely hampers the predictive accuracy for certain classes. Furthermore, as an array of sequence encoding methods continue to evolve, there has been a corresponding increase in model complexity. Addressing these issues, we introduce a novel Efficiency and Specificity-Based (ESB) class rebalancing strategy designed to mitigate the impact of class imbalance. Additionally, we assess the influence of six encoding schemes and four distinct architectural approaches on the prediction performance, employing four benchmark datasets for validation. Building upon these insights, we have developed a new hybrid model, termed CRISPR-MCA. Our experimental results demonstrate that the ESB strategy significantly surpasses the performance of existing baseline methods across multiple models. Moreover, the CRISPR-MCA model exhibits robust performance on two distinct types of datasets, affirming its effectiveness in enhancing the accuracy of deep learning predictions for off-target activities.

## Introduction

The CRISPR-Cas9 system, a transformative gene editing tool, has become the preferred method in various domains including genomics and biomedical research, owing to its efficiency, versatility, and precision [1, 2]. This system is composed of three fundamental components: CRISPR sequences (Clustered Regularly Interspaced Short Palindromic Repeats), the Cas9 protein (CRISPR-associated protein 9), and guide RNA (gRNA) [3]. CRISPR sequences, functioning as a genomic archive, store DNA fragments from invading viruses. In case of a subsequent viral attack, these sequences are transcribed into RNA that, together with the Cas9 protein, forms a ribonucleoprotein (RNP) complex, guiding Cas9/gRNA complex to identify onto viral sequences within the genome via complementary base pairing. Cas9, an RNA-guided DNA endonuclease, precisely cleaves the DNA’s double-helix structure at specific sites [4]. The resultant double-strand breaks (DSBs) trigger the cell’s intrinsic repair mechanisms, leading to targeted gene knockouts, corrections, or insertions [5–7].

As CRISPR-Cas9 technology advances, it brings forth novel prospects in genetic engineering. However, a critical challenge constraining its development is the issue of off-target effects. Due to the CRISPR-Cas9 system’s tolerance for mismatches of up to three bases between the gRNA and genomic DNAs [8, 9], gRNA can occasionally bind to DNA sequences that partially, rather than fully, match their intended targets. This non-specific binding results in the Cas9 acting upon and cleaving genomic sites beyond the designated target, potentially causing unintended genetic alterations [9]. Addressing this off-target problem is essential for enhancing the precision and safety of CRISPR-Cas9 applications in various fields [10].

Current methodologies for predicting off-target activity of CRISPR-Cas9 can be categorized into two principal approaches: experimental detection and in silico prediction [11]. The experimental approaches include two common methods. The first is the cell-free method, which operates independently of the cellular nuclear environment. In this approach, DNA is extracted from cells and subjected to nuclease reactions in vitro, facilitating the assessment of genomic cleavage effects. Notable techniques in this category include SITE-seq (selective enrichment and identification of tagged genomic DNA ends by sequencing) [12] and CIRCLE-seq (circularization for in vitro reporting of cleavage effects by sequencing) [13], among others that similarly bypass the cellular context. The second approach involves cell-culture methods, which consider the nuclear environment’s effects and provide a more comprehensive insight into the CRISPR-Cas9 system’s behavior within the cellular context. These methods include intricate procedures like WGS (Whole Genome Sequencing) [14], GUIDE-seq (genome-wide unbiased identification of DSBs by sequencing). While each of these experimental assays has its advantages, they also present limitations, including high cost, lengthy cycle times, and experimental complexity. Increasingly, researchers are adopting the simpler and more efficient in silico method for detecting off-target activity.

In silico approaches for predicting off-target activities in CRISPR-Cas9 systems are broadly classified into three main categories based on their underlying principles and methodologies [11]. The initial category, Methods based on Manual Rules, rely on predefined rules and heuristics derived from empirical data and expert knowledge. These methods primarily focus on sequence features, particularly the number and positions of mismatches between the gRNA and target DNA. For instance, the MIT Score method predicts off-target sites by considering both their location and count [6]. Similarly, the CROP-IT Score evaluates adjacent mismatch penalties, integrating heuristic evaluations based on the location of these mismatches [15]. In contrast, the CCTop Score method concentrates on the proximity of mismatches to the Protospacer Adjacent Motif (PAM) region, highlighting a different aspect of potential off-target interactions [16]. However, the accuracy can be limited due to the reliance on simplified assumptions and the inability to capture complex patterns in the data.

The second approach for predicting CRISPR-Cas9 off-target activities utilizes traditional machine learning techniques. In this method, a variety of algorithms are applied to learn from hand-crafted features within gRNA-target DNA sequences [17]. Generally, these approaches surpass the performance of rule-based methods. For example, CRISTA considers the potential for gene bulges and integrates multiple features to predict the likelihood of a specific gRNA binding to a genomic locus [18]. Another method, Elevation, evaluates individual guide-target pairs and compiles these assessments into a comprehensive guide score [19]. Despite their simplicity and expedited training duration, traditional models often fall short in accurately capturing the intricate spatial and sequence dependencies inherent in gene sequences, which are crucial for precise off-target prediction.

In recent years, deep learning techniques have been widely used in bioinformatics, to address the limitations of traditional methods in effectively leveraging complex information from gRNA-target DNA sequence pairs and large datasets, deep learning was increasingly employed in predicting CRISPR-Cas9 off-target activities [20–22]. Lin et al. [23] were the first to apply a deep learning model, introducing the CNN_std model, which used one-hot encoding to transform gRNA-target DNA into a 4*23 matrix using a Convolutional Neural Network (CNN). Liu et al. [24] developed the AttnToMismatch_CNN model, employing a word embedding technique for data encoding and combined CNN with Transformer models to predict off-target activities. In another advancement, Lin et al. [25] proposed CRISPR-Net, which innovated a new 7*24 size encoding scheme and was the first to consider off-target activities involving insertions or deletions in gRNA and target DNA sequence pairs. Guan et al. [26] designed a novel 14*23 size coding scheme, which, for the first time, accounted for noise in the original off-target dataset, further enhancing the model’s predictive accuracy.

Despite considerable advancements in model architecture and coding techniques within the realm of deep learning for CRISPR-Cas9 off-target activity prediction, a predominant challenge persists: high-class imbalanced data [27]. The datasets for off-target detection originate from whole-genome detection technologies. A critical issue arises, as these datasets identify significantly fewer off-target sites compared to potential mismatch sites. This discrepancy creates an imbalance between positive and negative samples leading to a biased learning process where models may tend toward overfitting in dominant categories. Previous researchers have employed various strategies to address this issue. For example, Chuai et al. [28] expanded the original data by randomly altering two bases in the distal region of the PAM, thus creating a new gRNA. However, this method may alter the specificity and efficiency of the original gRNA. Liu et al. [24] employed resampling to balance the samples. However, Zhang et al. [29] performed a systematic evaluation of both undersampling and resampling methods, discovering that these techniques could introduce more significant challenges than the original class imbalance issue and fail to improve model accuracy. Additionally, Charlier et al. [30] attempted to increase the number of positive samples by rotating the RNA-DNA encoded matrix by 90, 180, and 270 degrees. However, the effectiveness of this approach remains unverified. Recent work by Chen et al. [31] has enhanced our understanding of the molecular mechanisms of gRNA-target DNA hybrids through interaction fingerprints. This advancement underpins our initial attempt at data rebalancing, which utilizes the biological properties inherent in sequence pairs. This approach marks a departure from the conventional random sampling rebalancing schemes that are based solely on the data itself.

To address these challenges and fill the existing gaps, we analyzed the location, type, and tolerance of base mismatches within gRNA-Target DNA sequences and propose a novel class rebalancing strategy based on target efficiency and specificity screening to attenuate the effect of class imbalance in off-target datasets. Subsequently, we explore various encoding techniques for gRNA-target DNA sequences through detailed experimentation to identify the most effective method for this task. Building upon these foundations, we then delve into the effectiveness of different model structures in off-target prediction and propose a hybrid network model, CRISPR-MCA. This model integrates a multi-scale convolutional neural network and a multi-head self-attention mechanism, capable of extracting salient information from gRNA-Target DNA interactions across multiple scales and integrating the features, specifically designed to CRISPR-Cas9 system.

## Results

### Research on sizes of the coding scheme

In the realm of off-target prediction, the process of sequence encoding presents a significant challenge for feature extraction from sequence information. The diversity of encoding methods poses a question regarding the efficacy of different encoding sizes for model-based feature learning. To address this, we investigated the optimal encoding size for gRNA-target DNA sequences by evaluating six distinct One-hot encoding sizes, ranging from 23*4 to 20*20. These encodings were tested on the Hek293t and D7 datasets using two advanced models, CRISPR-MCA and CRISPR-IP, to ascertain the effectiveness of each encoding scheme in feature representation.

The experimental findings, as presented in Table 1, indicate that on the Hek293t dataset, CRISPR-MCA achieved the highest PR_AUC value of 0.7626 on coding scheme C2, removing the worst coding effect of C5, which is 11.3% higher than C4 and 0.6% higher than C3. Additionally, the recall value reached 0.6807, surpassing the sub-optimal result from C4 by 11.6%. Although its ROC_AUC value did not surpass the best-performing C3 encoding method, the difference was minimal, and it’s noteworthy that C3’s encoding size also is 24*7. Furthermore, CRISPR-IP recorded the highest PR_AUC (0.7233) and ROC_AUC (0.9926) values across two encoding schemes, C2 and C3, both with an encoding size of 24*7. The PR_AUC for C2 was superior to the sub-optimal C5, by 0.6% and significantly outperformed C1 by 17.2%. Similarly, C3’s ROC_AUC was 7% higher than that of C4.

**Table 1:** Performance of different models applying different coding schemes on two datasets.

			Hek293t			D7		
Model	Encoding	Size	Recall	ROC_AUC	PR_AUC	Recall	ROC_AUC	PR_AUC
CRISPR-MCA	C1	23*4	0.622	0.992	0.707	0.169	0.946	0.315
	C2	23*7	**0.681**	0.988	**0.763**	0.174	**0.963**	**0.386**
	C3	24*7	0.642	**0.992**	0.758	**0.295**	0.949	0.381
	C4	23*8	0.610	0.991	0.685	0.156	0.864	0.166
	C5	23*14	0.669	0.987	0.723	0.180	0.944	0.341
	C6	20*20	0.270	0.950	0.365	0.059	0.910	0.120
CRISPR-IP	C1	23*4	0.152	0.993	0.617	0.000	0.889	0.116
	C2	23*7	0.497	0.986	**0.723**	0.000	**0.980**	**0.435**
	C3	24*7	0.497	**0.993**	0.673	**0.171**	0.980	0.396
	C4	23*8	0.360	0.924	0.503	0.000	0.771	0.143
	C5	23*14	**0.582**	0.985	0.719	0.000	0.929	0.305
	C6	20*20	0.114	0.950	0.289	0.000	0.847	0.129

Note: C1 represents a 23*4 coding scheme utilized in the CNN_std model [23]; C2 and C3 both employ a 23*7 coding scheme, in the CRISPR-NET and CRISPR-IP models respectively [25, 29]; C4 adopts a 23*8 coding scheme [30]; C5 utilizes a 23*14 coding scheme in the latest CrisprDNT model [26]; and C6 features a 20*20 coding scheme within the DL-CRISPR model [32]. Note: C1 represents a 23*4 coding scheme utilized in the CNN_std model [23]; C2 and C3 both employ a 23*7 coding scheme, in the CRISPR-NET and CRISPR-IP models respectively [25, 29]; C4 adopts a 23*8 coding scheme [30]; C5 utilizes a 23*14 coding scheme in the latest CrisprDNT model [26]; and C6 features a 20*20 coding scheme within the DL-CRISPR model [32]. All results are averages of 5-fold cross-validation, where bold is the highest value. Additionally we modified the inputs of the corresponding models to accommodate different sized codes.

On the D7 dataset, CRISPR-MCA achieved the highest PR_AUC of 0.3864 and the highest receiver ROC_AUC of 0.9628 with the encoding scheme C2. It also achieved the highest recall of 0.2947 with encoding scheme C3, outperforming other encoding sizes. Additionally, experiments with CRISPR-IP on various encoding schemes corroborated these findings, with the 24*7-size encodings proving superior. Specifically, C2 attained a PR_AUC of 0.4347, which was 42.7% higher than that of C5, which has an encoding size of 23*14. It is noteworthy that in the D7 dataset, the recall value for all five encodings was zero. Upon analysis, this outcome may be attributed to the dataset’s composition, where the number of positive samples is only 52, significantly fewer than the number of negative samples. This imbalance likely causes the CRISPR-IP model to predict the majority class predominantly.

In summary, within the same model and dataset, a larger encoding size does not necessarily yield better results. For instance, the DL-CRISPR model’s C6 encoding, which augments the encoded matrix with 12 channels to denote mismatch types, adds 240 new units (20*12). However, as each encoding utilizes only 12 units, the feature matrix becomes overly sparse, with a utilization rate of merely 5%. This sparsity may introduce substantial noise into the model’s feature learning process. Conversely, the minimalistic C1 encoding, which focuses solely on the direct complementary pairing information between gRNA and Target DNA, neglects other sequence pair information such as mismatch positions’ relative relationship and the context of surrounding sequences. When designing a one-hot encoding schema, it is crucial to consider the richness of information in the matrix. The encoding should minimize noise and incorporate the effects of mismatch type and position based on biological mechanisms. It should highlight critical information such as the proximity of mismatch positions to the PAM region and the types of mismatches (e.g., transitions versus transversions), ensuring these elements are adequately represented in the encoding scheme.

### Effectiveness of different models in off-target prediction

This section is divided into two parts: initially, we assess the performance of distinct modules within the CRISPR-MCA model in predicting off-target effects, using two distinct datasets, K562 and D9, as our basis. Subsequently, we conduct a thorough examination of how various complex models influence off-target predictions. Table 2 outlines modifications to the original model for evaluation purposes: M1 involves the removal of Multi-CNN Layers, M2 pertains to the elimination of the Multi-Head Self-Attention mechanism, and M3 describes the exclusion of positional encoding. Owing to the significant impact of RNN and Transformer technologies in the field of NLP, these methodologies have also been adopted by researchers for off-target prediction applications. We substituted the original Multi-CNN Layers with BiLSTM to create model M4. Additionally, we replaced these with BiGRU in model M5, and introduced a more complex structure by incorporating the Transformer component in model M6. We also explored model M7, which combines the BiLSTM and Transformer modules, and model M8, which parallelizes these two modules. The complexity of models M4 through M8, as detailed in part (B), progressively increases. All the models maintain the inclusion of Dense layers in their architecture.

**Table 2:** (A) Models M1 to M3 are derivative models of the original model, each omitting three critical components. (B) Models M4 to M8 are designed with increasing levels of complexity, each composed of different complex modules. These models were all evaluated using the K562 and D9 datasets.

Model	K562			D9		
	Recall	ROC_AUC	PR_AUC	Recall	ROC_AUC	PR_AUC
(A)						
CRISPR-MCA	**0.740** ± **0.086**	0.987 ± 0.017	**0.802** ± **0.100**	**0.280** ± **0.160**	0.972 ± 0.021	**0.323** ± **0.070**
M1	0.588 ± 0.099	**0.996** ± **0.002**	0.765 ± 0.094	0.240 ± 0.163	0.976 ± 0.014	0.299 ± 0.130
M2	0.557 ± 0.188	0.983 ± 0.021	0.752 ± 0.169	0.180 ± 0.075	**0.981** ± **0.012**	0.220 ± 0.061
M3	0.564 ± 0.092	0.992 ± 0.005	0.741 ± 0.119	0.180 ± 0.075	0.980 ± 0.011	0.302 ± 0.087
(B)						
M4	0.583 ± 0.119	0.991 ± 0.009	0.745 ± 0.121	0.260 ± 0.150	0.940 ± 0.036	0.299 ± 0.095
M5	0.669 ± 0.144	0.996 ± 0.003	0.799 ± 0.121	0.140 ± 0.040	0.984 ± 0.010	0.256 ± 0.044
M6	0.646 ± 0.173	0.989 ± 0.015	0.775 ± 0.102	0.160 ± 0.196	0.961 ± 0.024	0.283 ± 0.131
M7	0.674 ± 0.100	0.988 ± 0.009	0.756 ± 0.138	0.160 ± 0.136	0.954 ± 0.023	0.238 ± 0.041
M8	0.631 ± 0.116	0.994 ± 0.003	0.721 ± 0.125	0.200 ± 0.063	0.976 ± 0.007	0.230 ± 0.061

All results are averages of 5-fold cross-validation, where bold is the highest value. Additionally we modified the inputs of the corresponding models to accommodate different sized codes.

In part A, it was observed that the omission of any component within the CRISPR-MCA model led to a decrease in evaluation metrics. Specifically, on the K562 dataset, the PR_AUC values for models M1-M3 diminished by 4.7%, 8.2%, and 6.6%, respectively, with an even more pronounced decline in the Recall values. In the D9 dataset, the reductions in PR_AUC were 8.2%, 7.2%, and 47%, respectively. Analysis of the variance in the three metrics’ results after five-fold cross-validation indicates that the full model’s stability surpasses that of the modified versions. Although the ROC_AUC was not optimal, the imbalanced nature of the data led us to prioritize PR_AUC outcomes over ROC_AUC in our comparisons. This suggests that our model, through its three distinct layers of modules, is adept at extracting complex information from gRNA-target DNA sequences.

In our findings detailed in part B, we noticed a trend: as models grow in complexity, PR_AUC values tend to drop across two datasets. In the D9 dataset, moving from the simpler M4 model to the more intricate M8 and M7 models saw PR_AUC values fall from 0.2992 to 0.2304 and 0.2378, showing declines of 29.9% and 25.8%, respectively. Intriguingly, within the K562 dataset, the M5 model’s performance closely mirrored that of CRISPR-MCA, with a negligible difference of only 0.2%. This highlights the BiGRU model’s competitive edge in capturing features from sequences with mismatches. Yet, the overall move towards more complex models doesn’t necessarily translate to improved predictions for off-target effects. This could be due to models overfitting, where they pick up on noise rather than meaningful patterns in the training data. Additionally, an overabundance of layers in Transformer models might dilute focus, spreading attention over irrelevant features and undermining the identification of crucial information.

Our study further supports Toufikuzzaman and colleagues’ assertion regarding the superior performance of simple models in predicting off-target effects [33].

### Comparison with state-of-the-art deep learning-based model

#### Model evaluation on mismatches-only datasets

To further assess our model, we initially compared it with six existing models using four mismatches-only datasets: D1, D2, D4, and D6. Datasets D1 and D2 were combined due to their common source, identical detection technique, and the insufficient size of D2 for independent evaluation.

**Fig 1:**
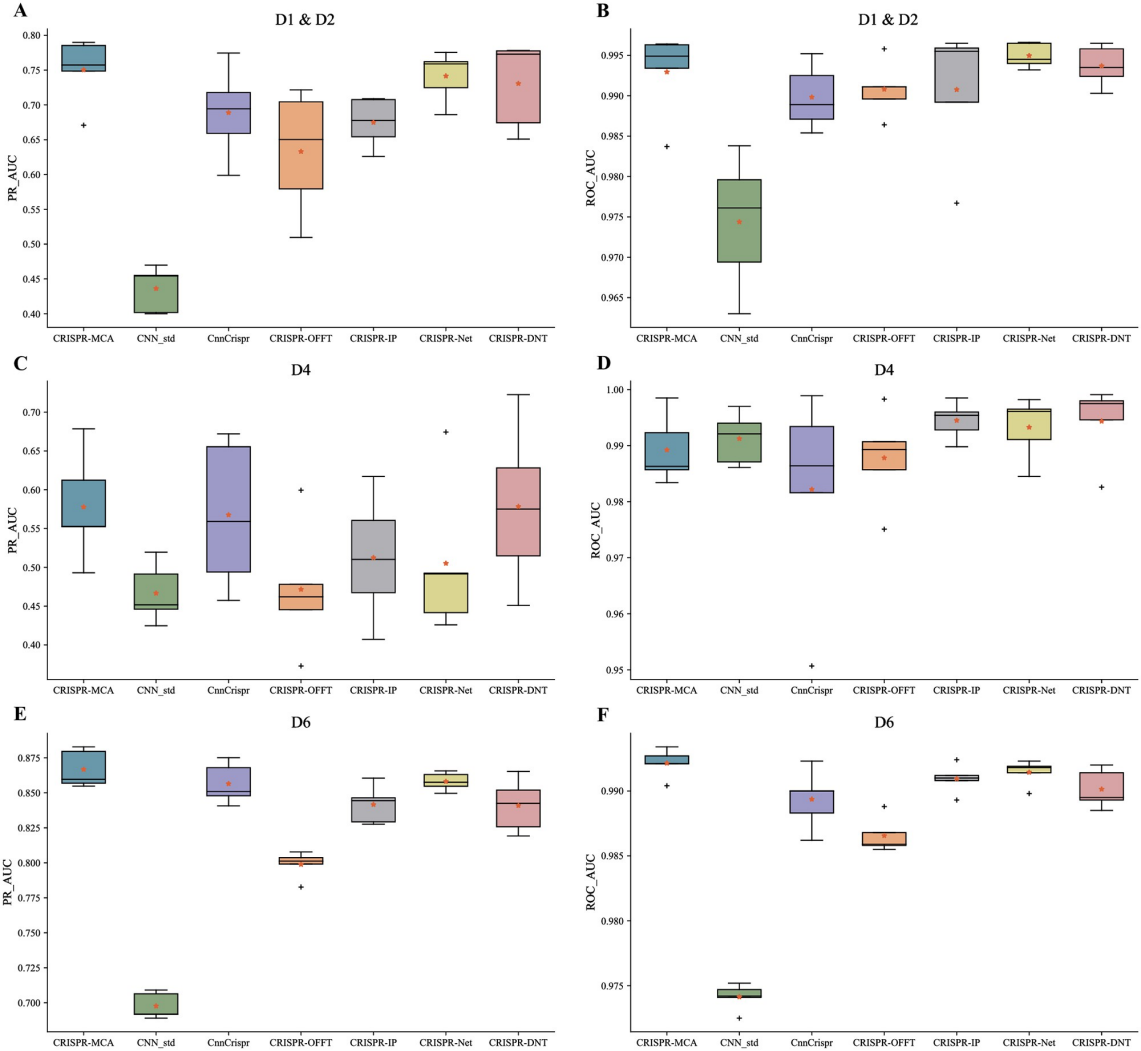
Comparison of the CRISPR-MCA model with six existing off-target prediction models on a mismatches-only datasets. **A,B** Performance of the seven models on the fusion datasets D1 (Hek293t) and D2 (K562), where A is PR_AUC and B is ROC_AUC. **C,D** Performance of seven models on dataset D4. **E,F** Performance of seven models on dataset D6.

According to the Box-plot presented in Fig 1, the CRISPR-MCA model surpasses the other six models on the combined D1 & D2 datasets, achieving an average PR_AUC of 0.7504 following 5-fold cross-validation. This performance is 2% superior to the most recent model, CRISPR-DNT (0.7308), and 11.1% better than CRISPR-IP (0.6749). CRISPR-DNT’s highest PR_AUC of 0.7898 also exceeds the peak performances of the competing models. Given that all models, with the exception of CnnCrispr, demonstrate an average Area Under the Receiver AUC_ROC value above 0.99, further analysis on AUC_ROC was deemed unnecessary. The PR_AUC and ROC_AUC metrics reveal that CRISPR-MCA exhibits a more compact box, indicating a narrower range of data variability across different folds and thus higher stability. In the D4, CRISPR-MCA demonstrated the second highest prediction performance, with an average PR_AUC of 0.5778. This is marginally lower than CRISPR-DNT’s 0.5783, yet represents a 12.7% enhancement over CRISPR-IP’s 0.5125. While CRISPR-MCA did not achieve the top performance in terms of the ROC_AUC, the difference between it and the leading model is minimal, amounting to only a 0.01% discrepancy. In a subsequent evaluation on dataset D6, CRISPR-MCA surpassed the other six models in both PR_AUC and ROC_AUC. CRISPR-MCA achieved an average PR_AUC of 0.8668, slightly exceeding the performances of CnnCrispr (0.8565), CRISPR-Net (0.8582), and CRISPR-DNT (0.8410), with improvements of 1.2%, 1%, and 3% respectively.

#### Model evaluation on mismatches and indels datasets

Furthermore, to validate our model’s competitiveness in analyzing data with both mismatches and indels (datasets D8 and D9), we conducted a comparison against three other models capable of off-target prediction for datasets with bulges. Models only capable of processing mismatches-only datasets were excluded from this comparison. Notably, the original framework of CRISPR-DNT does not support the input of 24-bit sequences. Therefore, we adapted the section of the model that processes input data to accommodate this requirement.

**Fig 2:**
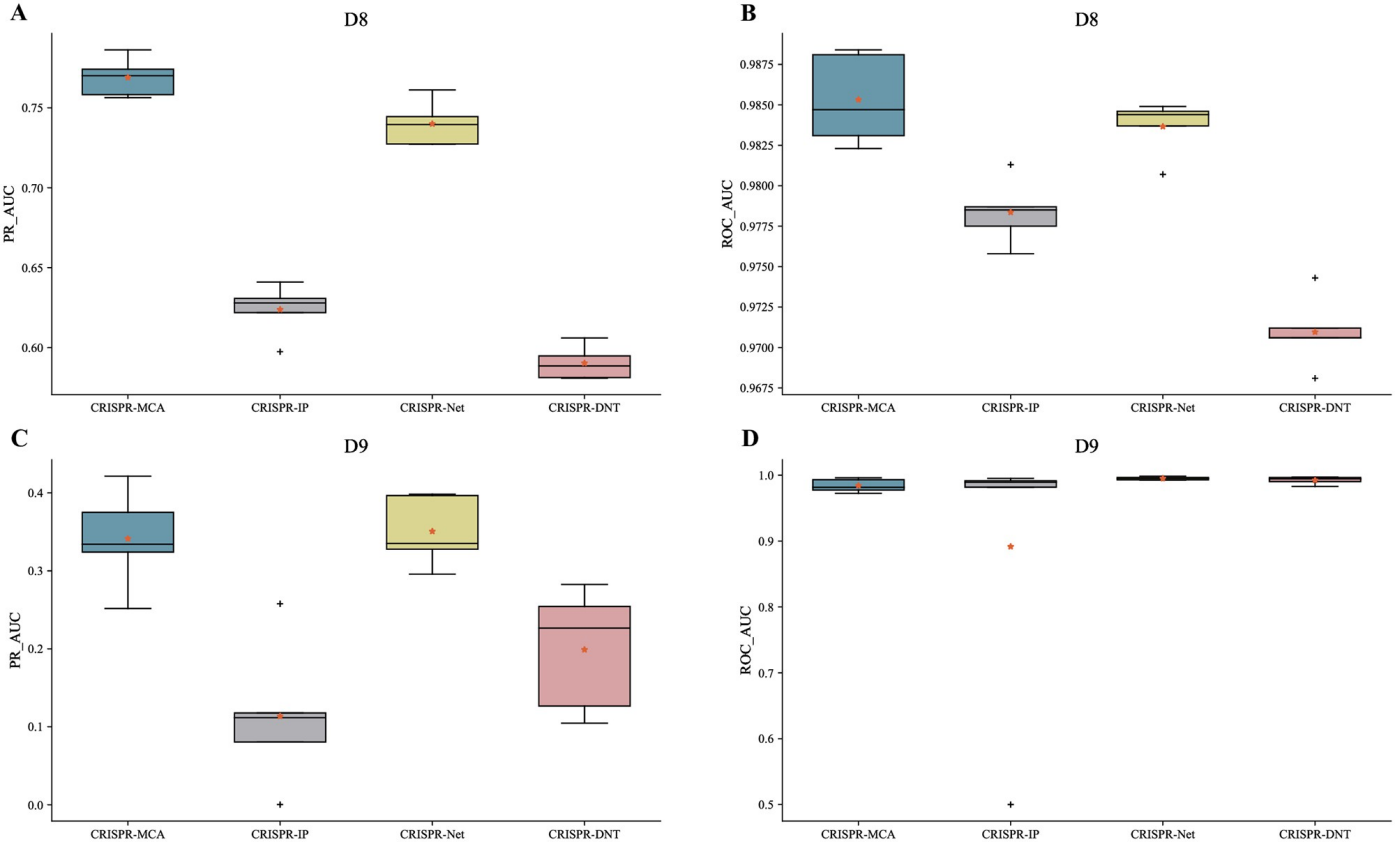
Comparison of the CRISPR-MCA model with six existing off-target prediction models on the included indels and mismatches datasets. **A,B** Performance of four models supporting indels and mismatches dataset on D8. **C,D** Performance of the four models on the D9 dataset.

Fig 2 illustrates that our CRISPR-MCA model demonstrates strong predictive capability on the moderately imbalanced dataset D8, achieving an average PR_AUC of 0.7691 and an ROC_AUC of 0.9853. This represents a 3.9% improvement in PR_AUC over the suboptimal CRISPR-Net. On dataset D9, although CRISPR-Net’s PR_AUC of 0.3507 slightly exceeds CRISPR-MCA’s 0.3413 by 0.009, and CRISPR-MCA does not achieve the highest ROC_AUC, the performance gap is narrow, maintaining our model’s competitive stance.

Drawing from the results of two independent experiments, it is evident that our model demonstrates competitive accuracy in off-target predictions across both available types of datasets, with notably superior performance in larger datasets.

### Efficiency and Specificity-based class rebalancing strategy

#### The level of class imbalance post-dataset rebalancing

Following the implementation of the ESB rebalancing strategy, there was a significant enhancement in the equilibrium of the datasets. Given that the IR, CVIR, and the IE metrics are all robust indicators of class imbalance, this analysis focuses solely on the IR for clarity. As detailed in Table 3, dataset D5 witnessed an approximately fivefold increase in positive sample count, from 56 to 281, accompanied by a reduction in the IR value from 6,846 to 1,364. Similarly, dataset D3, previously characterized by severe imbalance, now approaches a mild imbalance classification, evidenced by an increase in valid positive samples to 330 and a decrease in the IR value from 1,773 to 290. Furthermore, the optimization efforts have shifted datasets D4 and Hek293t from moderate to mild imbalance status, with their positive sample counts rising to 3,381 and 4,935, respectively. After the expansion, the mismatches-only datasets, namely D7, K562, and D6, have achieved a balanced state.

**Table 3:** The level of class imbalance within the dataset subsequent to the implementation of ESB rebalancing strategy.

Dataset	Positive	IR	Imbalance
D6	3767 → 41412	56.800 → 5.167	Balanced
K562	120 → 1076	168.325 → 18.772	Balanced
D7	52 → 327	193.789 → 30.817	Balanced
Hek293t	536 → 4935	246.974 → 26.824	Mild
D4	354 → 3381	831.017 → 87.010	Mild
D3	54 → 330	1773.611 → 290.227	Moderate
D5	56 → 281	6846.554 → 1364.438	Severe

#### Feature and validity of newly generated positive samples

**Fig 3:**
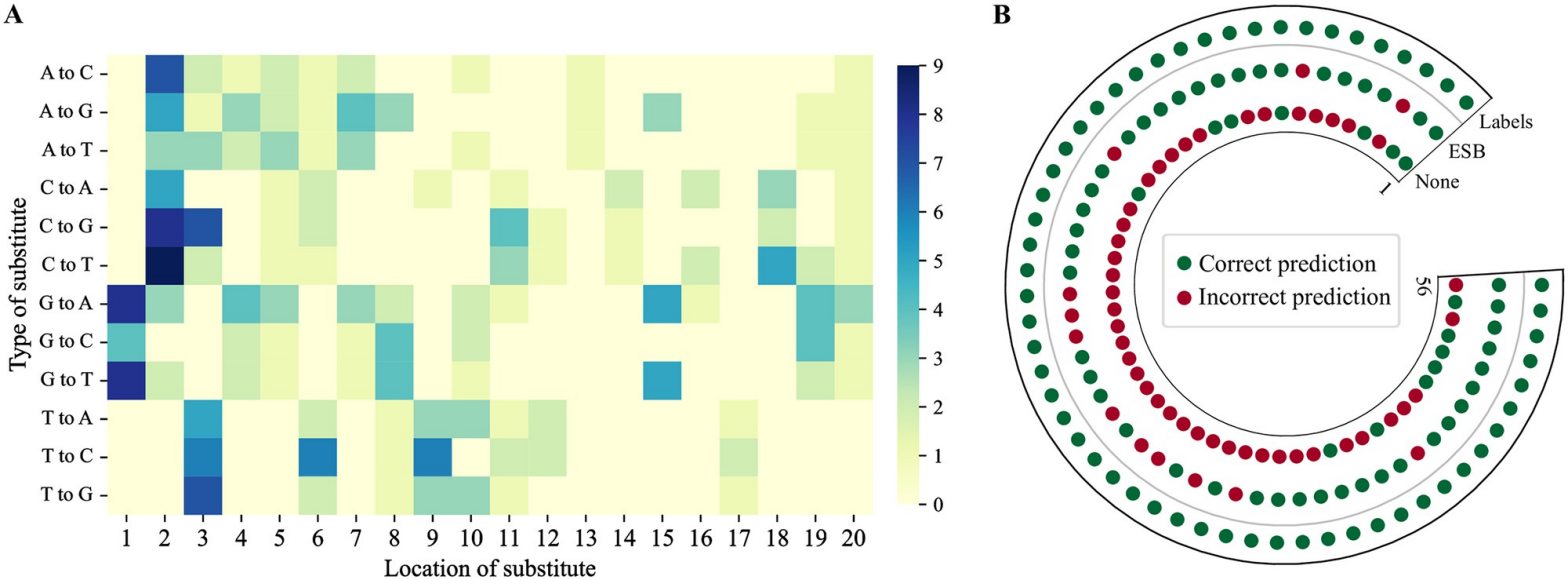
**A** The new samples feature heatmaps of the severely imbalanced D5 dataset, delineating the positions and types of nucleotides substituted in the generated positive samples of gRNA-target DNA. **B** Validation of positive off-target sites in dataset D5 predicted by rebalanced CRISPR-MCA. The outermost layer is the real sample, the middle layer is the prediction result after using ESB rebalancing strategy, and the innermost layer is the original prediction result. Incorrect and correct predictions are indicated in red and green, respectively.

Firstly, to verify the consistency of positive samples generated by our ESB rebalancing strategy with the original dataset’s inherent characteristics, we undertook a thorough analysis of all validly expanded positive samples. Fig 3A presents a heatmap showcasing the distribution and types of base substitutions within the D5 dataset’s expanded positive samples. This examination confirmed that the amplification data’s mismatch characteristics and specificity align with those of the original dataset. Notably, the sites of new gRNA substitutions are predominantly located in the sequence’s region distal to the PAM, especially at positions 1 to 3, mirroring the mismatch-tolerant sites in CRISPR-Cas9 mechanisms. Furthermore, it was observed that at position 1, substituting base G enhances gRNA editing efficiency, whereas substitutions of bases A, C, and T are less effective. At position 2, substituting T yields suboptimal outcomes, whereas alterations involving the other three bases demonstrate improved results. Additionally, the substitution of G for A and T has shown to yield favorable outcomes in off-target predictions.

Subsequent analysis, illustrated in Fig 3B, involved extracting positive samples from the severely imbalanced D5 dataset to compare prediction outcomes by CRISPR-MCA both with and without the application of a ESB. The findings indicate that the model, once rebalanced, demonstrated superior performance in predicting positive samples, accurately forecasting 44 out of 56, equating to a success rate of 78.6%. Conversely, the model lacking dataset rebalancing managed to correctly predict merely 14 out of 56 positive samples, resulting in a 25% accuracy rate.

#### Comparison and validation of effectiveness of rebalancing strategies

In this subsection, we evaluate the performance of the CRISPR-MCA model, employing the ESB strategy, against other methodologies across two severely imbalanced datasets, D3 and D5, to underscore our strategy’s efficacy in rectifying imbalances within off-target datasets. Notably, the domain of deep learning class rebalancing in this field is characterized by a paucity of techniques, with Upsampling and Class Weight Adjustment emerging as the predominant methods. Additionally, SMOTE and its variants show potential in addressing class imbalances to a certain degree. For comparative analysis, all methods were trained utilizing identical default parameters, and Table 4 encapsulates the outcomes via the mean of five-fold cross-validation results. Specifically, within the D3 dataset, the challenge of an exceedingly low count of positive samples renders all methods, barring our ESB strategy, ineffective at generating valid positive samples. This deficiency introduces significant noise into model training, detrimentally impacting outcomes compared to those achieved with the original dataset. Our ESB approach attained a PR_AUC value of 0.3766, marking a 32.6% enhancement from the baseline dataset. Compared to other methods, this represents a 37.6% and 36.5% improvement over Upsampling and ADASYN, respectively. In the case of the D5 dataset, while the impact is less pronounced than in D3, the ESB strategy still achieved a 6% improvement relative to the baseline, optimizing the PR_AUC value from 0.1717 to 0.2322. This adjustment also constitutes a 6% enhancement over the next most effective method, Upsampling, with a PR_AUC of 0.1722.

**Table 4:** CRISPR-MCA assesses six class rebalancing strategies on two datasets that exhibit severe imbalance. The table presents average outcomes obtained through 5-fold cross-validation.

Methods	D3	D5
None	0.2841	0.1717
Upsample	0.2737	0.1722
SMOTE	0.2555	0.1484
Borderline SMOTE	0.2525	0.1618
Class Weight Adjustment	0.2282	0.0022
ADASYN	0.2758	0.1632
ESB (Ours)	**0.3766**	**0.2322**

To further assess the ESB strategy across various models, we implemented ESB within six pre-existing models on the D5 dataset for off-target prediction experiments. Table 5 reveals that the application of ESB consistently enhances model performance, notably increasing the PR_AUC values of CRISPR-IP, CRISPR-OFFT, and CnnCrisp significantly, alongside marked improvements in the models’ accuracy in identifying positive samples. While the PR_AUC enhancement for CRISPR-DNT is less pronounced, its Recall value experiences a significant uplift from 0.1164 to 0.1764, constituting a 34.01% improvement and substantially bolstering the model’s overall capability. It is critical to highlight that the original dataset’s models often registered a Recall of 0, likely due to an insufficient quantity of positive samples impeding the models’ ability to learn from positive sample features. Post-ESB expansion, however, Recall values uniformly returned to normal, underscoring the ESB strategy’s effectiveness in mitigating the challenges posed by limited positive samples.

**Table 5:** Comparison of predictions from six existing models after implementing the ESB strategy.

MOdel	Recall	AUC_ROC	PR_AUC
CRISPR-DNT	0.1164	**0.9966**	0.1985
CRISPR-DNT_ESB	**0.1764**	0.9953	**0.2021**
CRISPR-Net	0.06	0.9952	0.1787
CRISPR-Net_ESB	**0.1182**	**0.9959**	**0.1830**
CRISPR-IP	-	0.9781	0.0819
CRISPR-IP_ESB	**0.4509**	**0.9820**	**0.1560**
CRISPR-OFFT	-	0.9737	0.0716
CRISPR-OFFT_ESB	**0.2527**	**0.9836**	**0.1282**
CnnCrispr	-	0.974	0.1147
CnnCrispr_ESB	**0.2545**	**0.909**	**0.2074**
CNN_std	-	**0.97**	**0.0973**
CNN_std_ESB	**0.1182**	0.9692	0.0955

In summary, the data samples generated by our proposed ESB strategy align closely with the characteristics of the original datasets. Specifically tailored for off-target datasets, ESB outperforms traditional class rebalancing methods. Our strategy represents a generalized approach, suitable for a broad spectrum of off-target prediction models addressing the challenges of imbalanced mismatches-only datasets.

## Discussion and conclusion

In recent years, the development of deep learning models for CRISPR-Cas9 off-target prediction in gene editing has seen rapid advancement. However, as research evolves, the diversity and complexity of sequence encoding approaches and proposed models have increased significantly. Moreover, these models often grapple with the challenge of dataset class imbalance, complicating the accurate prediction of off-target sites. To address these issues, we conducted an in-depth analysis of the biological properties of gRNA-target DNA sequences, emphasizing the significance of base-pairing preferences and tolerance. We reviewed and experimented with various One-hot encoding schemes to identify the most effective encoding method for capturing the essential information of sequences. Our findings suggest that the dimensionality of encoding schemes must strike a balance: too small, and vital information is lost; too large, and excessive noise hampers the model’s feature learning. An optimal encoding size, approximately 24*7, was identified as it minimizes information loss while preventing the introduction of unnecessary noise, thereby enhancing model performance.

Furthermore, the adaptation of large-scale models from the field of NLP to off-target prediction has led to increased model complexity. However, our findings indicate that such complexity does not necessarily enhance performance in off-target prediction tasks. In response, we introduce a hybrid network model, CRISPR-MCA, which leverages a multi-scale convolution and multi-head attention mechanism for feature extraction. Our experimental results demonstrate that CRISPR-MCA effectively extracts relevant features from sequences, delivering superior performance across two distinct types of datasets when compared to existing models.

Class imbalance represents a significant challenge for current off-target prediction models, leading to predictions that disproportionately favor the majority class due to a paucity of positive samples. To counteract this issue, we introduce, for the first time, an Efficiency and Specificity-Based (ESB) class rebalancing strategy. Notably, this strategy uniquely employs biological characteristics to augment the dataset, diverging from traditional methods that rely on repeated sampling of the original dataset. Our experimental analyses demonstrate that the ESB rebalancing strategy effectively mitigates the challenges posed by imbalanced datasets during model training, outperforming conventional rebalancing methods. When applied across various models and severely unbalanced datasets, the ESB strategy consistently delivers superior performance. This underscores its potential as a generalized approach for mismatches-only datasets, highlighting its promising applicability in the field.

Limitation of this study: As highlighted in our study, the Efficiency and Specificity-Based (ESB) rebalancing strategy is specifically tailored for datasets characterized by mismatches-only off-target datasets, which are the most common source of off-target phenomena. Nevertheless, this strategy is not applicable to datasets that include indels, owing to the presence of bulges which hinder the computation of off-target scores and the statistical analysis of potential off-target sites across the genome. Additionally, the potential for noise introduction into the dataset post-rebalancing has not been addressed in our current methodology. Moving forward, our research will addressing these challenges, with the aim of continuously improving the precision of off-target effect predictions, thereby enhancing the overall utility and safety of CRISPR-Cas9 technologies.

## Materials and methods

### Datasets

In this research, we compiled two distinct types of benchmark datasets for our experimental and analytical purposes. Of these, seven datasets were composed solely of mismatches, while the remaining two encompassed both mismatches and indels. These datasets were derived from various off-target site detection methods and include gRNAs, target DNAs, and labels. Inactive off-target sites are labeled as ‘0’, while validated off-target sites are marked as ‘1’, as shown in Table 6. In the first type of dataset, the Hek293t and K562 datasets include more than 150,000 inactive off-target sites from different human cells identified using the bowtie2 assay, alongside 30 gRNAs and 656 validated off-target sites. Datasets 3, 4, and 5 consist of off-target datasets obtained through the GUIDE-Seq assay from Kleinstiver et al. [34], Tasi et al. [35], and Listgarten et al. [19], respectively. Dataset 3 features 5 gRNAs and encompasses over 90,000 off-target sequence pairs. Dataset 4 includes 9 gRNAs and nearly 300,000 off-target sites, while Dataset 5 comprises 22 gRNAs with approximately 400,000 potential sites. Dataset 6, constructed by Cameron et al. [12], contains 3767 validated off-target sites identified by SITE-Seq across 9 gRNAs. Dataset 7 encompasses 19 gRNAs and was validated using multiple techniques, including PCR, Digenome-seq, and HTGTS. In the second type, dataset 8 represents the initial dataset containing indels, proposed by Tsai et al. through the CIRCLE-seq assay [35]. It comprises 500,000 targets associated with 10 gRNAs. Dataset 9 contains more than 200,000 pieces of data related to 6 gRNAs.

**Table 6:** Details of the two types of datasets utilized in the experiments and analyses.

Type	Dataset	Detection method	gRNAs	Positive	Negative	Source
Mismatches-only	Hek293t	Digenome-seq, BLESS, and others	18	536	132378	chuai et al. [28]
	K562	Digenome-seq, BLESS, and others	12	120	20199	chuai et al. [28]
	D3	GUIDE-Seq	5	54	95775	Kleinstiver et al. [34]
	D4	GUIDE-Seq	9	354	294180	Tasi et al. [35]
	D5	GUIDE-Seq	22	56	383407	Listgarten et al. [19]
	D6	SITE-Seq	9	3767	213966	Cameron et al. [12]
	D7	PCR, Digenome-Seq and HTGTS	19	52	10077	Haeussler et al. [36]
Mismatch and indel	D8	CIRCLE-seq	10	7371	577578	tsai et al. [13]
	D9	GUIDE-Seq	6	50	213883	Listgarten et al. [19]

### Analysis of the degree of data imbalance

The substantial size of the seven mismatches-only off-target datasets employed in this study, encompassing approximately 900,000 items, the proportion of positive samples is relatively small, comprising around 4,000 items. The remaining category comprises two datasets, collectively encompassing approximately 800,000 items, of which only 7,431 are positive samples, with the majority localized within D8. The datasets we examined are significantly challenged by class imbalance, a critical issue highlighted by our calculations of the degree of imbalance using three metrics: Imbalance Ratio (IR), Coefficient of Variation of the Imbalance Ratio (CVIR), and Information Entropy (IE). These metrics are crucial for identifying and understanding the inherent challenges and biases in these datasets.

In this study, IR is defined as the ratio of the number of Negative Samples to the number of Positive Samples within a dataset. A higher IR value indicates a more pronounced class imbalance. CVIR is utilized to evaluate the variability of the imbalanced data. An elevated CVIR value signifies greater variability in the degree of class imbalance within the dataset. It is defined as follows:

$$\begin{array}{c} CV_{IR}=\frac{\sigma_{IR}}{\mu_{IR}} \end{array}$$ (1)

where *σ*_*IR*_ is the standard deviation of the imbalance ratio and *μ*_*IR*_ is the mean of the imbalance ratio.

Information entropy serves as a quantitative measure of the proportional imbalance between positive and negative samples in a binary dataset. It reaches its maximum value when the dataset is perfectly balanced, meaning the number of positive and negative samples is equal. Conversely, in cases where the dataset is highly unbalanced (i.e., one category significantly outweighs the other in sample count), the information entropy will be notably lower. The respective mathematical calculations for this metrics are elucidated as follows:

$$\begin{array}{c} H\left(X\right)=-p\;{\text{log}}_{2}\left(p\right)-q\;{\text{log}}_{2}\left(q\right) \end{array}$$ (2)

where *p* and *q* are the proportions of positive and negative samples in the datasets, respectively.

The Table 7 presents the outcomes of our analysis. The datasets exhibit an average IR of 1:1607, with the maximum and minimum IRs recorded at 1:6846 and 1:56, respectively. Furthermore, the CVIR exceeds 0.96 across the board, predominantly surpassing 0.99, while all the IE values remain below 0.12. From these findings, we classify the datasets into categories of imbalance severity. Specifically, datasets K562, D6, D7, and D8, with an IR below 200, a CVIR between 0 and 0.99, and an IE higher than 0.04, are designated as mildly imbalanced. Hek293 and D4, which have an IR ranging from 200 to 1000, a CVIR not exceeding 0.998, and an IE within 0.01 to 0.04, are considered moderately imbalanced. The remaining datasets D3, D5, and D9 are classified as severely imbalanced due to their distinct metrics.

**Table 7:** Analysis of the degree of imbalance between positive and negative samples in the datasets, where the Total is the number of all samples in the dataset, the IR stands for Imbalance Ratio, the CVIR denotes the Coefficient of Variation of the Imbalance Ratio, and IE signifies Information Entropy.

Dataset	Total	IR	CVIR	IE	Imbalance
D6	217733	56.8001	0.9654	0.1260	Mild
D8	584949	78.3582	0.9747	0.0975	Mild
K562	20319	168.3250	0.9882	0.0522	Mild
D7	10129	193.7885	0.9897	0.0464	Mild
Hek293t	132914	246.9739	0.9919	0.0379	Moderate
D4	294534	831.0169	0.9976	0.0134	Moderate
D3	95829	1773.6111	0.9989	0.0069	Severe
D9	213933	4277.67	0.9995	0.0031	Severe
D5	383463	6846.5536	0.9997	0.0021	Severe

### Mismatch features and specificity analysis

**Fig 4:**
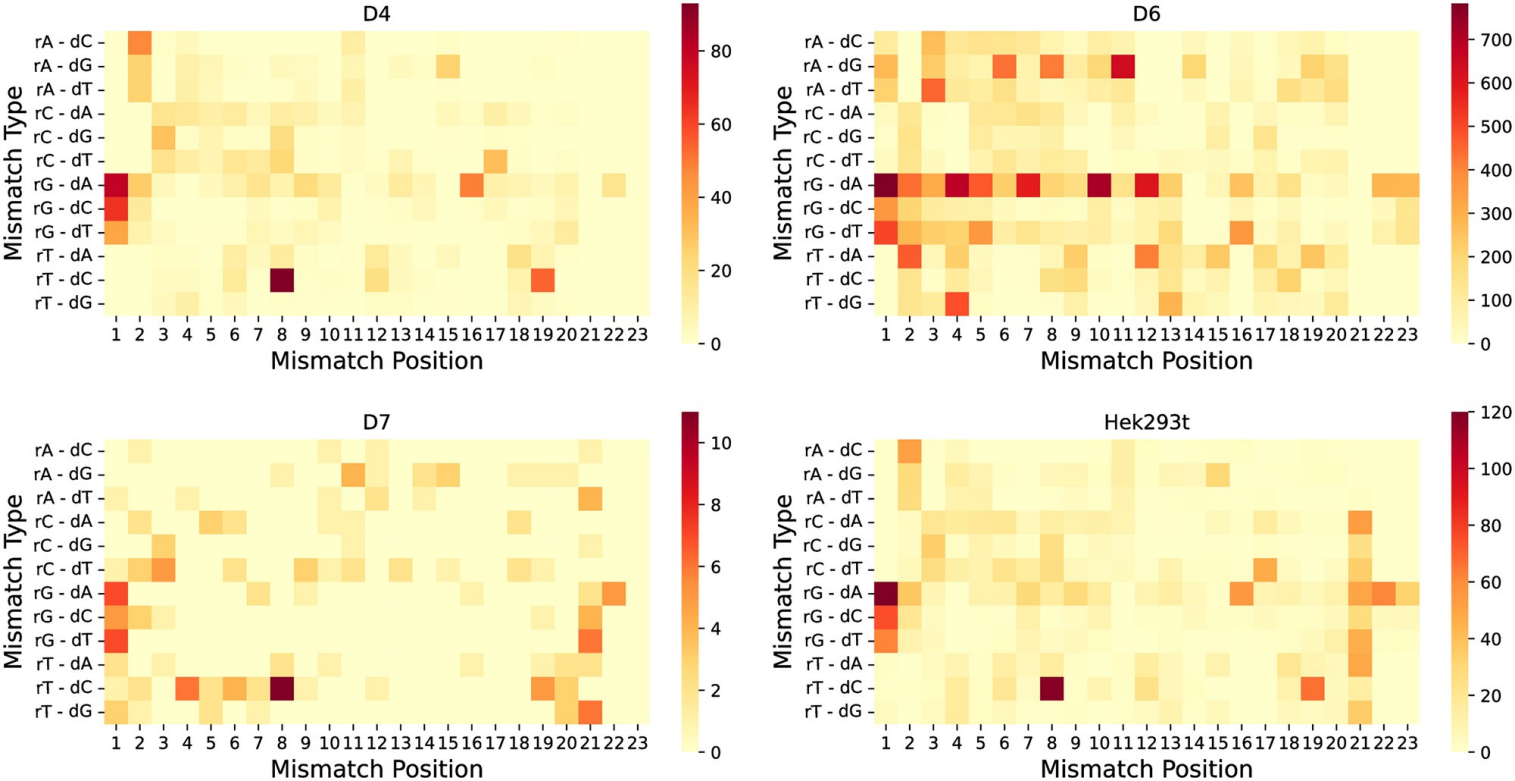
The heatmaps illustrate the types of base mismatches occurring at various positions across the four datasets.

To comprehensively investigate the influence of base mismatch types on off-target activity, we analyzed the distribution of mismatch types across various positions in off-target positive samples, which can give us insights into the mechanism of the bases [37]. These samples were not included in the D8, D9 datasets due to the inclusion of bulges. This analysis incorporated a range of detection techniques, with a particular focus on the HEK293t cell line and the GUIDE-Seq detection employed in the D4 dataset, among others. Excluding positions 21-23 (the NGG region), our findings, as depicted in Fig 4, revealed a notable impact of the G-A mismatch, particularly at positions 1 and 16. Additionally, we observed significant effects of T-C mismatches at positions 8 and 19, G-C at position 1, and both G-T and A-C mismatches at position 2, on off-target activities. These observations align with the previous research [26]. Intriguingly, in the D6 dataset, which has a large sample, we identified various mismatch types contributing to off-target effects. This could be attributed to the SITE-Seq assay’s tolerance for a higher number of mismatched bases. However, these results generally corroborate the trends noted in other datasets.

**Fig 5:**
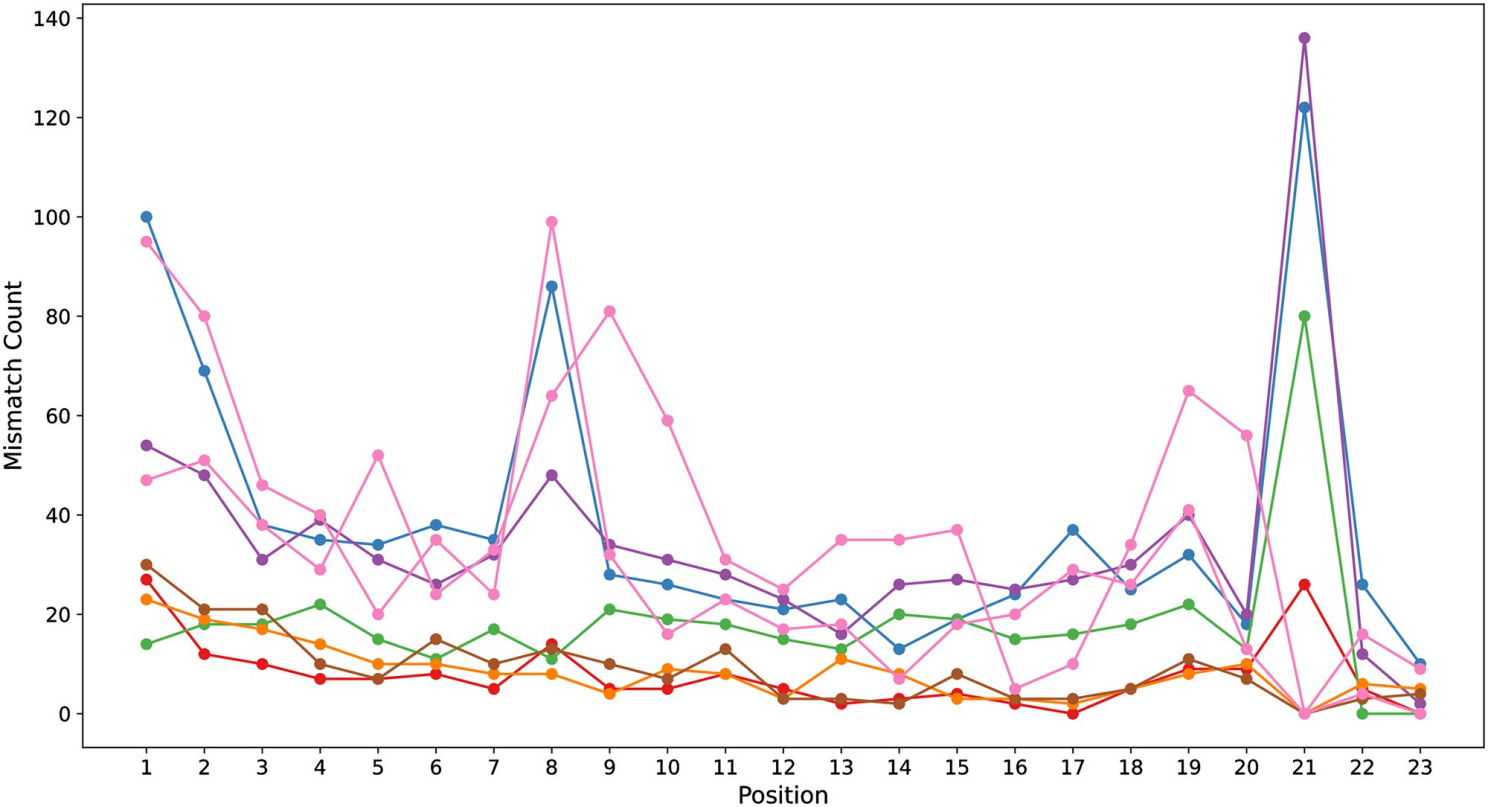
The frequency of mismatches at varying positions within gRNA-target DNA sequences across multiple datasets.

We extended our research to examine the locations of mismatches within the gRNA-target DNA sequences and observed that base mismatches at various positions exert distinct effects on off-target activity. Fig 5 illustrates our analysis, where we quantified clip mismatches at each position across all positive samples in the dataset. Notably, we found a lower tolerance for mismatches in the proximal region of the Protospacer Adjacent Motif (PAM), located at positions 21-23. This region exhibited fewer mismatches between positions 10-20 compared to other sequence regions. In contrast, the distal region of the PAM showed an increased frequency of mismatches. Moreover, positions 1, 2, 8, and 9 recorded a significantly higher number of mismatches than other regions. These findings are in alignment with those of previous studies [4, 38]. The insights gleaned from these base pairing dynamics are instrumental in our subsequent data augmentation strategy, enabling the generation of new gRNA variants with enhanced specificity and efficiency.

### Encoing gRNA-target DNA sequences

In this study, we used datasets that included both gRNA sequences and their corresponding DNA sequences from potential off-target sites. The gRNA sequences consisted of the nucleotide bases of adenine (A), guanine (G), cytosine (C), and uracil (U), while DNA sequences consisted of the nucleotide bases of adenine (A), cytosine (C), guanine (G), and thymine (T). To maintain the consistency of the gRNA sequence with the DNA sequence, uracil (U) is replaced by thymine (T) in the gRNA. Drawing on recent advances in the field, the two most effective coding strategies currently recognized are One-hot coding and word embedding techniques.

In recent research, the One-hot coding technique has been shown to be effective in capturing features between gRNA and DNA sequences by Lin et al. and Guan et al. [25, 26]. To implement this coding scheme, we assign two types of channels to represent the raw data: bases channel and direction channel. In the base channel, five different One-hot vectors represent the four nucleotide bases and indels (Insertions and deletions) as follows: adenine is coded as [1, 0, 0, 0, 0], guanine is coded as [0, 1, 0, 0, 0], cytosine is coded as [0, 0, 1, 0, 0], thymine is encoded as [0, 0, 0, 1, 0], and indels indicated by underscores (_) are encoded as [0, 0, 0, 0, 1]. This coding strategy transforms the gRNA and DNA sequences of length 24 into two-dimensional matrices, each with dimensions 24*5. In the foregoing section, it is demonstrated that the nature of mismatches significantly influences off-target effects. Consequently, we integrate the two matrices using the XOR operation, thus enabling a structured and detailed representation of the genetic information. However, this approach has a limitation: the inability to express directional nuances of base mismatches or deletions. For example, the coding of ‘GA’ and ‘AG’ combinations produces the same representation [1, 1, 0, 0, 0], which may result in a loss of information during the learning phase of the model. To address this issue, we introduce two directional channels dedicated to encapsulating the directional information of these base interactions. With these improvements, we successfully extend the coding of gRNA-target DNA pairs into a more comprehensive 24*7 matrix format.

**Fig 6:**
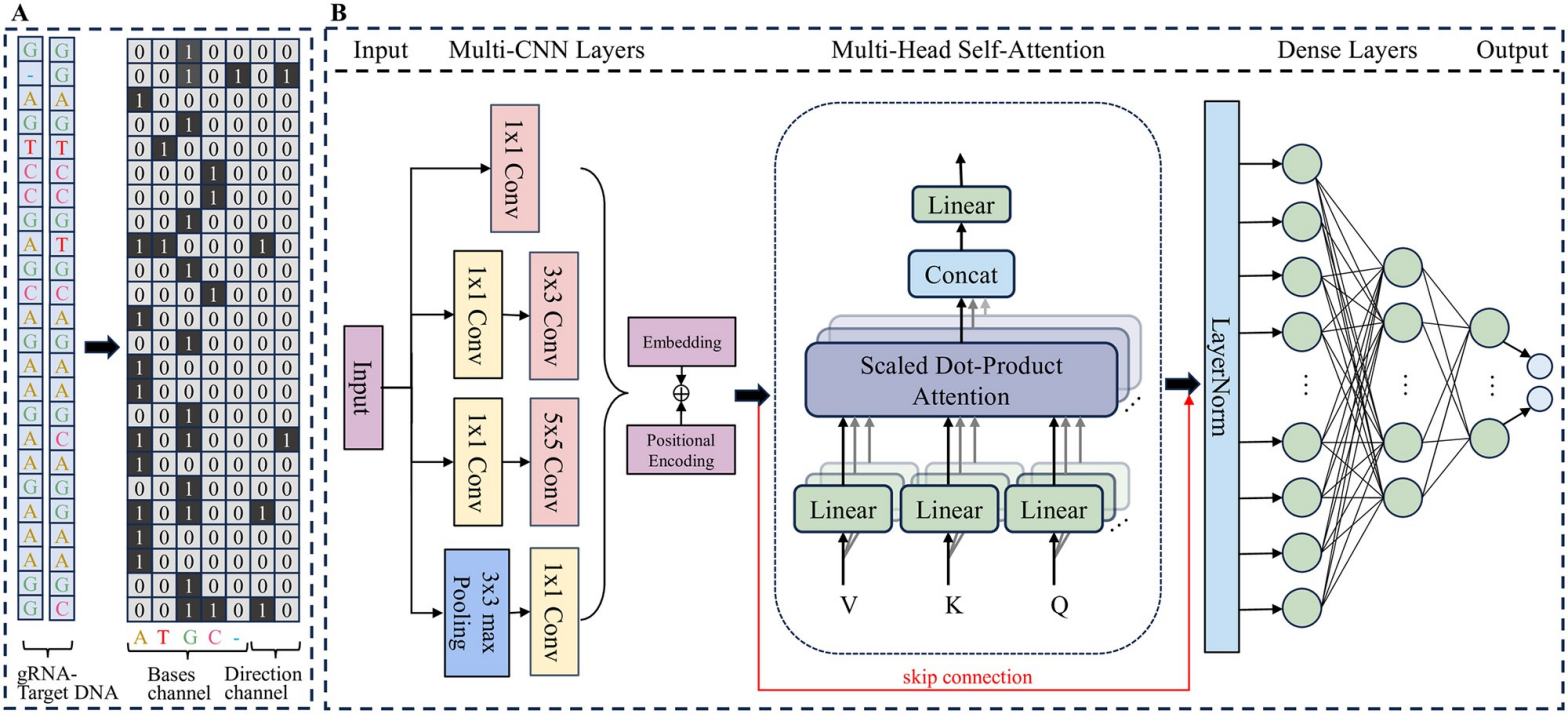
**A** Example of a gRNA-target DNA sequence code. The first five channels are base channels, which are responsible for converting base pairs in the sequence into unique One-hot vectors, adenine is coded as [1, 0, 0, 0, 0], guanine is coded as [0, 1, 0, 0, 0], cytosine is coded as [0, 0, 1, 0, 0], thymine is encoded as [0, 0, 0, 1, 0], and indels indicated by underscores (_) are encoded as [0, 0, 0, 0, 1]. The last two channels are direction channels, which are used to mark the direction of the bases at the position where the mismatch occurred. **B** The architecture of CRISPR-MCA is depicted, starting with a 24*7 matrix derived from the encoded gRNA-target DNA sequence as its input. This matrix is processed by a multiscale convolutional layer designed to extract sequence features. The output from the Multi-CNN Layers undergoes positional encoding before being input into the Multi-Head Self-Attention layer for further sequence analysis. Following processing, the data is merged with earlier inputs and then channeled through three dense layers, comprising 256, 128, and 2 neurons respectively. The final layer utilizes a softmax activation function to yield binary classification outcomes.

As illustrated in Fig 6A, the base channel encoding for the bulge ‘_-G’ base pair is represented as [0, 0, 1, 0, 1], while its orientation channel is encoded as [0, 1]. For the mismatches-only base pair ‘A-T’, the base channel is encoded as [1, 1, 0, 0, 0], with the orientation channel represented by [1, 0]. The ‘G-G’ base pair’s base channel is encoded as [0, 0, 1, 0, 0], and its direction channel is encoded as [0, 0], reflecting the mismatch direction.

### The network architecture of CRISPR-MCA model

Off-target gRNA-DNA interactions frequently exhibit complex patterns that markedly diverge from the linguistic structures typically analyzed in the natural language processing (NLP) domain [39]. To address this challenge, we introduce CRISPR-MCA, a state-of-the-art hybrid deep learning model specifically designed for predicting off-target activity. This innovative model combines multi-scale convolution with a multi-head attention mechanism, enabling sophisticated multi-feature extraction and fusion. This integration is crucial for capturing the unique biological signals inherent in genomic off-target sequences, providing a nuanced understanding of mismatches and insertions/deletions (indels) between gRNAs and target DNAs. As illustrated in Fig 6B, the network architecture of CRISPR-MCA can be broadly divided into six parts: (1) Input Layer, (2) Multi-CNN layer, (3) Positional Encoding Layer, (4) Multi-Head Self-Attention Layer, (5) Dense Layer, and (6) Output Layer.

The Input layer of the model receives the coding matrix from the gRNA-target DNA sequence pair with a size of 24*7. This matrix is then forwarded to a Multi-CNN layer for processing and this layer comprises four parallel branches: a 1 × 1 convolution, a 1 × 1 convolution followed by a 3 × 3 convolution, a 1 × 1 convolution followed by a 5 × 5 convolution, and a 3 × 3 maximum pooling succeeded by a 1 × 1 convolution. The original matrix is initially downscaled using a 1 × 1 convolution kernel to lessen computational demands. Subsequently, various convolution kernels are employed for initial feature extraction. The outcomes are then concatenated to achieve fusion of features across different scales. This architecture is designed to enable the model to discern both local and global information pertaining to bases within the input matrix at varying scales and resolutions, thereby enhancing the comprehensive analysis of gRNA-target DNA interactions.

Following feature extraction, the model reshapes and normalizes the data. Given that our data comprise sequences, the position and order of base pairs are paramount. The positional encoding layer is adept at incorporating information from all other positions in the sequence while processing the bases at each position. This mechanism significantly enhances the model’s ability to comprehend the context of the sequence, thereby improving its accuracy in predicting off-target effects. The position encoding formula is as follows: for even positions of the matrix (i.e., i is even), the formula for the sine function is applied:

$$\begin{array}{c} PE_{\left(pos,2i\right)}=\text{sin}(\frac{pos}{10000^{2i/d_{\text{model}}}}) \end{array}$$ (3)

For odd positions of the matrix (i.e., i is odd), the formula for the cosine function is utilized:

$$\begin{array}{c} PE_{\left(pos,2i+1\right)}=\text{cos}(\frac{pos}{10000^{2i/d_{\text{madel}}}}) \end{array}$$ (4)

where *pos* is the position index in the sequence, *i* is the index of the dimension, and *d*_*model* is the model dimension. Here $10000^{2i/d_{\text{model}}}$ is a scaling factor used to adjust the frequency according to the dimension *i* so that the position encoding can vary at different frequencies, allowing the model to capture the position information efficiently even in very long sequences.

The position-encoded data is processed through the Multi-Head Self-Attention (MHA) mechanism, which projects the input queries (Q), keys (K), and values (V) into distinct representational spaces through linear transformations. This mechanism executes parallel attention operations within these spaces and subsequently integrates their results. Our feature analysis reveals the complexity of the sequence information between gRNA and target DNA, encompassing base pairing as well as mismatches, bulges, and the protospacer adjacent motif (PAM). This approach enables our model to focus on various segments of the gRNA-target DNA sequence pairs at different positions, capturing the intricate relationships among these elements to precisely pinpoint potential off-target sites. To configure its upper and lower layers, we established a total of eight heads. Additionally, to prevent gradient issues due to a high number of heads, we implemented skip connection, a strategy that permits the direct transmission and amalgamation of position-encoded data with the output from the MHA mechanism.

Finally, the output from the MHA mechanism is flattened and subsequently passed through three dense layers containing 256, 128, and 2 neurons, respectively. The first two layers employ the ReLU activation function to introduce non-linearity. To combat model overfitting, dropout regularization was implemented with a rate of 0.5. The final dense layer produces the probability of off-target activity.

### Efficiency and Specificity-Based class rebalancing strategy

**Fig 7:**
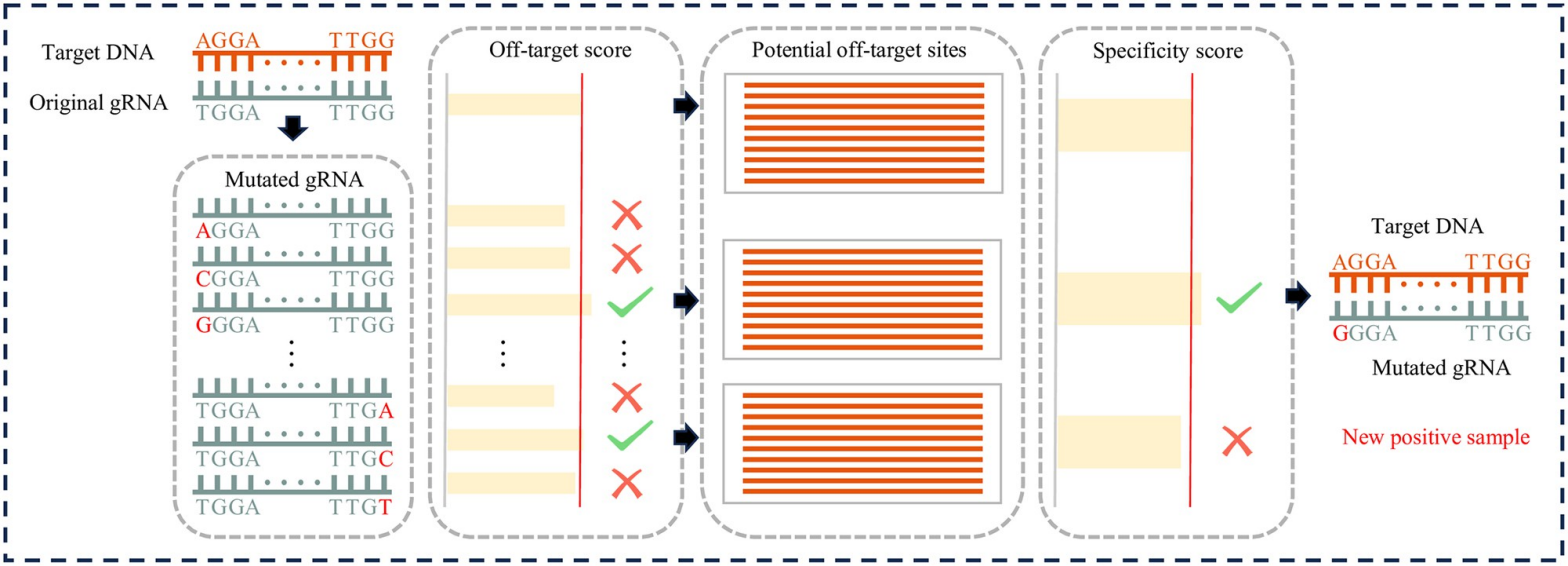
The Efficiency and Specificity-Based class rebalancing strategy encompasses two distinct phases: Initial mutation screening of gRNAs and subsequent specificity assessment of high-efficiency mutants. Initially, each nucleotide within a gRNA is substituted with the three alternative nucleotides, following which the targeting efficiencies of these mutated gRNAs are evaluated. Mutants demonstrating superior efficiency compared to the original gRNA are selected for further analysis. The second phase involves identifying potential off-target sites across the genome using Cas-Offinder, succeeded by calculating the specificity of mutant gRNAs’ interaction with the target DNA sequence. Mutants that exhibit specificity surpassing that of the original sequence are then adopted as enhanced positive samples for subsequent training purposes.

We are motivated by the findings from the mismatch features and specificity analysis, it was evident that each nucleotide alteration in the positive sample gRNAs could potentially impact off-target effects. We introduce a novel strategy, termed Efficiency and Specificity-Based class rebalancing (ESB), aimed at augmenting the positive sample within the mismatches-only dataset. This approach is elucidated in Fig 7, the method comprises two distinct phases: the mutation of gRNAs followed by the specificity screening of these mutations for enhanced efficiency.

Initially, acknowledging the potentially minimal impact of singular mismatches on targeting efficiency, we manipulated each nucleotide across the 20 positions of the gRNA sequence, substituting it with the three other nucleobases. This process yielded 60 novel mutated gRNAs per original sequence. Given the propensity of these mutations to alter the original gRNAs’ targeting efficiencies, we employed the CRISOT-Score methodology, as proposed by Chen et al. [31], for the assessment of off-target scores of each mutated gRNA. CRISOT-Score is a novel off-target scoring function that utilizes RNA-DNA interaction features to assess the scores. This function incorporates key interaction characteristics and SHAP (SHapley Additive exPlanations) scores to evaluate the impact of each base pair [40]. The scoring is refined by aggregating these values according to base pair type and spatial location, enabling precise scoring of specific base pair types at defined locations. Variants demonstrating higher off-target scores than the original sequence were earmarked for subsequent analysis. Notably, the CRISOT-Score, integrating 193 molecular characteristics per base pair within the gRNA-target DNA alignment, facilitates a comprehensive quantification of off-target activities. The formula is as follows:

$$\begin{array}{c} S_{RD,i}=\sum_{j=1}^{N}\text{mean}([{V_{i}^{F_{RDj}}}_{1},{V_{i}^{F_{RDj}}}_{2},\ldots ,{V_{i}^{F_{RDj}}}_{n}]) \end{array}$$ (5)

$$\begin{array}{c} S=a\sum_{i=1}^{20}S_{RD,i}+b \end{array}$$ (6)

where *RD* is the base pair on gRNA and DNA, *F*_*RDj*_ is the *j* significant feature of this bp, and ${V_{i}^{F_{RDj}}}_{n}$ is the *F*_*RDj*_ feature score at the *i* position. Finally, *a* and *b* are used to map the result to the range [0, 1].

Upon developing more efficient gRNAs, we observe an increase in the potential for active off-target effects and variations in specificity. Therefore, we carry out the second phase of processing. In this phase, the gRNAs pinpointed in the initial stage undergo sequencing. This is succeeded by an exhaustive genome-wide search utilizing Cas-Offinder, set to a maximum mismatch tolerance of 1, aimed at identifying all potential off-target sites for variant gRNAs [41]. We then calculate the specificity score for each variant gRNA-target DNA pair. The gRNA-target DNA pairs that demonstrate both high efficiency and specificity are selected as the new positive training samples for subsequent research.

To prevent data leakage during testing, rebalancing will be exclusively conducted on the training dataset. Considering the possibility of mutated gRNAs duplicating those in the test data, any identical gRNAs in the rebalanced dataset will also be removed. Finally, de-duplication operations will be conducted on both the test and training datasets.

### Selection of the baseline model

In this study, we rigorously evaluated the representativeness, diversity, and performance benchmarks of existing CRISPR-Cas9 off-target prediction models, ultimately selecting six models for detailed analysis. As shown in Table 8, CNN_std is a pioneering model that leverages deep learning techniques to predict off-target effects in CRISPR technologies, representing a significant advancement in the field. The models CRISPR-OFFT and CnnCrispr are distinguished by their novel use of word vector encoding, making them the first in this arena to adopt such a methodology. CRISPR-Net is the first off-target prediction model that takes into account indels in gRNA-Target DNA sequences. CRISPR-IP, on the other hand, utilizes a combination of CNN, BiLSTM, and an attention mechanism to adeptly learn features from sequence pairs. This model amalgamates the strengths of previous off-target prediction models, incorporating an extensive array of feature extraction modules from diverse deep learning architectures. In a distinctive approach, CRISPR-DNT employs a dual-layer, full transformer architecture, marking it as the first of its kind in off-target prediction. These models not only epitomize the forefront of CRISPR technology applications but also serve as robust benchmarks. Their efficacy has been substantiated on publicly available datasets, providing a solid foundation for comparing new models and ensuring that our findings are both generalizable and representative.

**Table 8:** Detailed information about the selected comparison models, specifically regarding their support for ‘Mismatches’ and ‘Indels’ within datasets. The types of datasets that the model can predict are labeled as ‘Supported’ and those that it cannot predict are labeled as ‘Unsupported’.

Model	Encoding Method	Mismatches	Indels	Source
CNN_std	One-hot encoding	Supported	Unsupported	[23]
CRISPR-OFFT	Word embedding	Supported	Unsupported	[42]
CnnCrispr	Word embedding	Supported	Unsupported	[25]
CRISPR-Net	One-hot encoding	Supported	Supported	[25]
CRISPR-IP	One-hot encoding	Supported	Supported	[29]
CRISPR-DNT	One-hot encoding	Supported	Supported	[26]

### Experimental design

Our experiments were conducted under a consistent training methodology, utilizing the same code environment and hardware. We employed TensorFlow 2.3.2 as the deep learning framework, with our computational resources comprising a Core i7-13700K processor, and 128G of RAM. To ensure the stability and reliability of our evaluation results, we adopted a 5-fold cross-validation approach for model training. In this method, the dataset is randomly partitioned into five equal-sized subsets. During each phase of validation, one subset serves as the validation set for assessing model performance, while the remaining four subsets are amalgamated to form the training set. Additionally, for the purpose of parameter optimization in model training, we allocated 10% of the validation set to function as a test set.

Given the diverse structures of different models, convergence times vary, rendering it inequitable to apply a uniform epoch across all models. Therefore, we set the default epoch count to 500, incorporating an EarlyStopping technique to prematurely conclude model training when the reference metrics remain unchanged for 10 consecutive epochs. Owing to the high-class imbalanced nature of our datasets, conventional evaluation metrics fall short of delivering an effective assessment. Consequently, we employ Recall, PR-AUC, and ROC-AUC as our evaluation metrics. ROC-AUC assesses a model’s overall performance across various thresholds, whereas PR-AUC is particularly apt for scenarios with imbalanced positive and negative samples, evaluating model performance through the area under the precision-recall curve. Our experiment consisted of the following five parts:

1. We explore the effectiveness of different coding schemes. The current landscape of coding schemes for off-target prediction is characterized by an ever-increasing complexity and diversity. Nevertheless, the efficacy of different one-hot encoding approach for gRNA-target DNA interactions remains a topic of debate. To address this, we have compiled a selection of the six most effective one-hot encoding schemes reported in the literature for examination. These include the 23*4 scheme and subsequent 24*7 scheme, by Lin et al. [23, 25], the 23*8 scheme by Charlier et al. [30], the 20*20 scheme by Zhang et al. [32], a different 24*7 scheme proposed by Zhang Li et al. [29], and the 23*14 scheme by Guan et al. [26].
2. Additionally, we explore the performance of various state-of-the-art models in off-target prediction tasks. These models include CNN, LSTM [43, 44], GRU [45, 46], Transformers [47, 48], and Attention mechanisms [49, 50], along with diverse combinations thereof.
3. Subsequently, we compare our newly developed model, CRISPR-MCA, against six existing models to showcase its enhanced capabilities in off-target prediction. The models evaluated include the recent CRISPR-DNT, as well as CNN_std, CnnCrispr, CRISPR-OFFT, CRISPR-Net, and CRISPR-IP.
4. Ultimately, we confirm the effectiveness of the ESB Rebalancing Strategy when applied exclusively to datasets with mismatches-only off-target instances, and we explore the imbalance trends observed within the augmented dataset. The class rebalancing strategies evaluated for comparison encompass the Synthetic Minority Over-sampling Technique (SMOTE) [51], which creates new samples by interpolating between a few instances of the minority class, and its derivatives, Borderline SMOTE [52] and ADASYN (Adaptive Synthetic Sampling) [53]. Additionally, commonly employed techniques such as Upsampling and Class Weight Adjustment are also considered.
